# Unraveling tumor specific neoantigen immunogenicity prediction: a comprehensive analysis

**DOI:** 10.3389/fimmu.2023.1094236

**Published:** 2023-07-25

**Authors:** Guadalupe Nibeyro, Veronica Baronetto, Juan I. Folco, Pablo Pastore, Maria Romina Girotti, Laura Prato, Gabriel Morón, Hugo D. Luján, Elmer A. Fernández

**Affiliations:** ^1^ Centro de Investigación y Desarrollo en Inmunología y Enfermedades Infecciosas (CIDIE), Consejo Nacional de Investigaciones Científicas y Técnicas (CONICET)/Universidad Católica de Córdoba (UCC) & Fundación para el Progreso de la Medicina, Córdoba, Argentina; ^2^ Facultad de Ingeniería, Universidad Católica de Córdoba (UCC), Córdoba, Argentina; ^3^ Universidad Argentina de la Empresa (UADE), Instituto de Tecnología (INTEC), Buenos Aires, Argentina; ^4^ Instituto Académico Pedagógico de Ciencias Básicas y Aplicadas, Universidad Nacional de Villa María, Villa María, Córdoba, Argentina; ^5^ Departamento de Bioquímica Clínica, Facultad de Ciencias Químicas, Universidad Nacional de Córdoba (UNC), Córdoba, Argentina; ^6^ Centro de Investigaciones en Bioquímica Clínica e Inmunología (CIBICI), Consejo Nacional de Investigaciones Científicas y Técnicas (CONICET), Córdoba, Argentina; ^7^ Facultad de Ciencias de la Salud, Universidad Católica de Córdoba (UCC), Córdoba, Argentina; ^8^ Facultad de Ciencias Exactas, Físicas y Naturales (FCEFyN), Universidad Nacional de Córdoba (UNC), Córdoba, Argentina

**Keywords:** immunotherapy, cancer immunology, neopeptide, immunogenic neoantigen database, immunoinformatic

## Abstract

**Introduction:**

Identification of tumor specific neoantigen (TSN) immunogenicity is crucial to develop peptide/mRNA based anti-tumoral vaccines and/or adoptive T-cell immunotherapies; thus, accurate in-silico classification/prioritization proves critical for cost-effective clinical applications. Several methods were proposed as TSNs immunogenicity predictors; however, comprehensive performance comparison is still lacking due to the absence of well documented and adequate TSN databases.

**Methods:**

Here, by developing a new curated database having 199 TSNs with experimentally-validated MHC-I presentation and positive/negative immune response (ITSNdb), sixteen metrics were evaluated as immunogenicity predictors. In addition, by using a dataset emulating patient derived TSNs and immunotherapy cohorts containing predicted TSNs for tumor neoantigen burden (TNB) with outcome association, the metrics were evaluated as TSNs prioritizers and as immunotherapy response biomarkers.

**Results:**

Our results show high performance variability among methods, highlighting the need for substantial improvement. Deep learning predictors were top ranked on ITSNdb but show discrepancy on validation databases. In overall, current predicted TNB did not outperform existing biomarkers.

**Conclusion:**

Recommendations for their clinical application and the ITSNdb are presented to promote development and comparison of computational TSNs immunogenicity predictors.

## 1 Introduction

Tumor specific neoantigens (TSNs) are unique antigenic peptides that emerge from genomic alterations covering single nucleotide variants (SNVs), nucleotide insertions or deletions, alternative splicing and/or gene fusion events (1–4), among others. These alterations may produce dysfunctional proteins by non-synonymous mutations, changes to the open reading frames as well as fusion proteins that may give rise to neopeptides at the junction (2, 3). These proteins, when processed by the proteasome, may generate different neopeptides that are presented on the cell surface bound to the MHC-I molecules (i.e., neoantigens), and be recognized by T cell receptors, thus potentially triggering an immune response (5). Among the current advances in immune checkpoint blockade immunotherapy (ICB), personalized neoantigen-based cancer vaccines have been shown to prime host immunity against tumor cells and are under clinical trials. Presently, neoantigen discovery comprises of integration of next generation sequencing (NGS), immunology and computational biology and screening the genomes/exomes/transcriptomes for somatic sequence changes that may produce putative neoantigens. After prioritization, if considered immunogenic, they are synthesized as mRNA, DNA or peptides and administered by vaccination. Therefore, improving identification of TSNs and definition of its formulation for delivering to the immune system are needed to increase tumor-specific T-cell responses and thus the benefit from current cancer ICB (6). The most complex step in neoantigen discovery processes is their computational prediction of immunogenicity, which is done by testing a repertoire of variants identified by NGS. As concluded by the European Society for Medical Oncology (ESMO), improvements in their identification, selection and prioritization are needed to increase the scope of benefit of cancer vaccines and adoptive T-cell therapies. Moreover, ongoing clinical trials will cast light over those cancer types and therapy combinations that could increase benefit from neoantigen-based immunotherapies (7). Currently, several computational pipelines for neoantigen prediction are available (8–12). Most TSNs immunogenicity predictors, if not all, are based on predictions of the peptide-major histocompatibility complex (p-MHC-I) binding affinity (BA) by software like NetMHCpan (13), MHCFlurry (14) or MixMHCpred (15) where BA is used as the main prediction for MHC-I presentation, but also as immunogenicity predictor. Recent approaches incorporate additional features like variant allele fraction, gene expression, and clonality of mutations. However, these concepts have led to an excessive number of putative neoantigens with low experimental immunogenicity validation rates, making their use impractical and financially ineffective for personalized vaccine development. Despite using predicted p-MHC-I affinity as the main concept, methods like PRIME (16), DeepImmuno (17) and DeepHLApan (18) calculate an immunogenicity score; the first one, through a logistic regression and the others including a deep learning based fine tuning immunogenicity step, trained over non-tumoral immunogenic peptides.

A thorough evaluation of existing immunogenicity prediction tools would require knowledge of surface-presented TSNs with proven MHC-I binding and positive or negative TCR recognition (immunogenic and non-immunogenic TSNs). Despite several attempts to develop useful tumor specific neoantigens databases, none of these databases make full use of existing information. For instance, Cancer Antigenic Peptide Database (CAPD) (19) and DbPepNeo (20), lack of truly non-immunogenic peptides, limiting the evaluation of false positive rates. The TANTIGEN database (21), incorporates tumor T cell antigens, but information regarding experimental MHC-I binding, validation method or immunogenicity classification is not clearly accessible. The caAtlas, containing tumor immunopeptidomics and incorporating neoantigens (22), lack information about peptide immunogenicity, making it impractical for predictor performance assessment. The TSNAdb (23) provides predicted neoantigens arising from SNVs in different cancer types, however, they are putative neoantigens without experimental validation thus it cannot be used as “the truth” in software evaluation. The NEPdb (24) is a promising database although not all of the immunogenic neoantigens are proved to be presented on cell surface (they may elicit an *in-vitro* immune response, but they would not be processed and consequently not presented). Another drawback of current neoantigens databases is that not all negative examples were proved to bind to an HLA. To mention, the CEDAR database (25), does not allow searching for epitopes that simultaneously meet positive MHC-I ligand assay with negative T cell assay. In the same way, in order to know if the neoantigen is processed and presented, a one by one analysis should be conducted or it requires choosing the “*in-vivo*” category, losing a big amount of data. Recently, the Tumor Neoantigen Selection Alliance to improve cancer vaccines and therapy through Artificial Intelligence, acknowledging the need of curated datasets to train better models, launched its TESLA neoantigen database (26), but the processing and presentation of their positive peptides were not experimentally validated. In addition, for the negative peptides, despite having experimentally measured binding affinity, they do not present the wild type (WT) sequence or the gene name, at least in its public version. Despite such database diversity and the lack of neoantigen cell processing, presentation and immunogenicity validation, several benchmark studies were conducted in recent years. For instance, Schaap-Johansen et al. (27) explored different algorithms by comparing results from the original publications, thus not allowing a fair comparison since each publication uses a different database. Buckley et al. (28) performed a comprehensive analysis of different immunogenicity predictors mainly affording the difference between pathogen and neoantigen immunogenicity using two different datasets: the TESLA database and an in-home glioblastoma HLA-A*02:01 neoantigens database, both without experimental validation of the neoantigen cell processing and presentation. Such heterogeneity of databases and approaches yields difficult to reach unbiased and comprehensive comparison in immunogenicity predictors/prioritizators.

**Figure 1:**
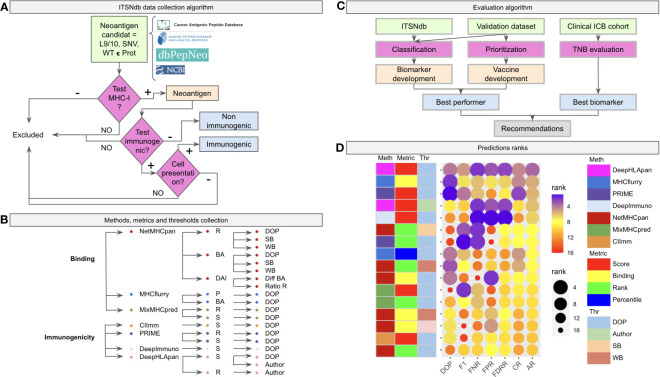
Benchmark workflow scheme. **(A)** The algorithm of inclusion criteria for the tumor specific neoantigens (immunogenic not immunogenic) into the ITSNdb. **(B)** The seven predictive software (three neoantigen binder predictors: netMHCpan, MHCFlurry and MixMHCPred, and four neoantigen immunogenicity predictors: CIImm, Prime, DeepImmune, DeepHLApan) with their associated metrics (R, Rank; BA, Binding Affinity; DAI, Differential Agretopicity Index; P, Percentile; S, Score) and thresholds (DOP, Distance to the Optimal Point; SB, Strong Binder; WB, Weak Binder; Author, Autor method suggested threshold) evaluated in the present study. **(C)** Evaluation pipeline with the used datasets and characteristics evaluated over each method. **(D)** Prediction performance for each method according to different performance metrics ranks. DOP, DOP rank; F1, F1-Score rank; FPR, False Positive Rate rank; FNR, False Negative Rate rank; FDRR, False Discovery Rates Rank; CR, Classification Rank; AR, Average Rank.

In order to fulfill this gap and to provide a comprehensive framework for immunogenic predictor/prioritizator evaluation, the following datasets are presented here: i) the new Immunogenic Tumor Specific Neoantigen database (ITSNdb), a manually curated neoantigen database, created using a novel approach, including only neoantigens meeting the following inclusion criteria (**Figure 1A**): (a) peptides derived from non-silent somatic SNVs with WT sequence, proved present in the referenced protein sequence; (b) with experimentally validated binding to the MHC-I molecules (by mass spectrometry method -MS- or through competition binding assays); (c) validated positive or negative immunogenicity reaction tested on immunogenic assays (i.e., tetramer titration and IFN-γ or TNF ELISPOT^®^) and d) positive and experimentally validated processing and presentation (by MS or through transfection of mutated genes into adequate Antigen-Presenting Cells) over immunogenic ones. As result the proposed ITSNdb enables the analysis of immunogenicity without the interference of BA and processing (already proven), being the main improvements against current TSN databases. ii) the use of data emulating true patient derived neoantigens scenario as a validation strategy for prioritization and iii) predicted neoantigens used to build the tumor neoantigen burden predictors from ICB cohorts with tumor mutation burden and outcome associations in order to evaluate the impact on biomarker definitions. Finally, non-immunogenic peptides from the TESLA database were used to further evaluate false positive rates, but left it out from the evaluation algorithm since the TSNs selection procedure is not reproducible in a clinical setting. Through this new database as well as the proposed validation datasets, a comprehensive immunogenicity predictor’s assessment was performed by using the most referenced and state of the art methods, elucidating the current challenges in predicting/prioritizing immunogenic tumor neoantigens for biomarker and vaccine development. Finally, we summarize the results, and highlight potential areas requiring future research.

## 2 Results

### 2.1 The Immunogenic Tumor Neoantigen database

The Immunogenic Tumor Neoantigen database was built, currently having 199 nine and ten-mer SNV-derived neoantigens with their WT counterparts, restricted HLA information, gene, tumor tissue, peptide length, mutation position type (i.e., MHC-I anchor position if the amino acid change is located at position 2 or 9 in the sequence or MHC-I non-anchor position otherwise) immunogenicity reaction and references (available at github/elmerfer/ITSNdb). They were curated from more than 70 publications based on the inclusion criteria depicted in **Figure 1A**, according to Riley et al. recommendations (29), who highlighted the necessity of knowledge about non-immunogenic epitopes that bind to MHC-I molecules but do not elicit a T cell response. Therefore, ITSNdb only includes neoantigens with MHC-I presentation and positive/negative immunogenic reaction experimentally validated, resulting in 129 immunogenic and 70 non-immunogenic TSNs. The HLA-A*02:01 represents 80/199 (40.2%) of the HLA restricted peptides (**Figure S1**).

### 2.2 A comprehensive comparison of seven software programs and their different metrics for immunogenicity prediction evaluated over ITSNdb, validation and clinical ICB cohorts databases design

Seven software programs (three neoantigen binding predictors and four immunogenic predictors) with their associated metrics and proposed thresholds, leading to 19 methods (**Figure 1B**), were evaluated as depicted in **Figure 1C**. The ITSNdb was used to calculate the classification performance metrics (see Statistical analysis section). In order to evaluate TSN prioritization, a validation dataset comprising 120 peptide-HLA pairs emulating a real patient scenario (i.e. total neoantigens predicted for a patient by means of RNAseq or whole exome sequencing), was used; and a set of 99480 predicted neoepitopes for TNB calculation from four different ICB cohorts with outcome association (30–33), were used for biomarker analysis (all the samples, data and code are available at github/elmerfer/ITSNdb).

**Figure 1D**, a graphical representation of **Table 1**, illustrates the performance of each method-metric-threshold predictor combination (**Figure 1B**) accounting for classification and prioritization (**Figure 1C**). Each combination is sorted considering the average ranks (AR) of the following performance metrics: DOP (Distance to the Optimal Point which accounts for performance, certainty and event incidence) (34), F1-score (which accounts for positive detection and certainty) calculated onto ITSNdb and the FPR and FNR (False Positive and Negative Rates respectively) calculated over the validation dataset. In addition, the Classification Rank (CR, average between DOP and F1-score ranks) and False Detection Rates Rank (FDRR, average over FPR and FNR ranks) are shown. It can be seen that DeepHLApan was the top performing method.

**Table 1:** Performance values calculated for each metric.

Method	Classification		ITSNdb			Validation		Ranks ITSNdb		Ranks Validation		Order	
	Metric	Thr	DOP	F1	auc	FPR	FNR	DOP	F1	FPR	FNR	AR	ARsd
DeepHLApan	I	DOP	0,88	0,69	0,57	23,89	28,57	4	5	3	2	3,5	1,29
MHCflurry	B	DOP	0,83	0,67	0,6	36,28	42,86	2	7	7	6	5,5	2,38
PRIME	SI	DOP	0,82	0,7	0,58	53,98	42,86	1	4	12	6	5,75	4,65
DeepHLApan	I	A	0,92	0,65	NA	23,89	28,57	9	10	3	2	6	4,08
DeepImmune	I	DOP	0,95	0,64	0,52	16,81	14,29	12	12	1	1	6,5	6,35
NetMHCpan	R	SB	1,04	0,71	NA	75,22	28,57	14	3	14	2	8,25	6,65
PRIME	RI	DOP	0,84	0,66	0,57	47,79	71,43	3	8	10	13	8,5	4,20
MHCflurry	P	DOP	0,88	0,68	0,53	49,56	71,43	4	6	11	13	8,5	4,20
NetMHCpan	R	WB	1,1	0,77	NA	95,58	28,57	15	1	16	2	8,5	8,10
NetMHCpan	B	DOP	0,88	0,61	0,57	23,01	85,71	4	13	2	16	8,75	6,80
MixMHCpred	S	DOP	0,92	0,61	0,52	34,51	57,14	9	13	6	10	9,5	2,89
MixMHCpred	R	DOP	0,93	0,66	0,54	57,52	42,86	11	8	13	6	9,5	3,11
NetMHCpan	B	WB	1,2	0,74	NA	75,22	42,86	16	2	14	6	9,5	6,61
NetMHCpan	B	SB	1,03	0,65	NA	38,05	57,14	13	10	8	10	10,25	2,06
CIImm	I	DOP	0,9	0,6	0,55	39,82	57,14	7	15	9	10	10,25	3,40
NetMHCpan	R	DOP	0,9	0,59	0,56	33,63	71,43	7	16	5	13	10,25	5,12

Thr, Threshold; DOP, Distance to the Optimal Point; FPR, False Positive Rate; FNR, False Negative Rate; AR, Average Rank; ARsd, Average Rank standard deviation; B, Binding; R, Rank; P, Percentile; I, Immunogenicity; S, Score; SB, Strong Binder; WB, Weak Binder; A, Authors’ proposed threshold; NA, Not Applicable.

### 2.3 A deep learning-based immunogenicity predictor ranked as the top performer

**Figure 2:**
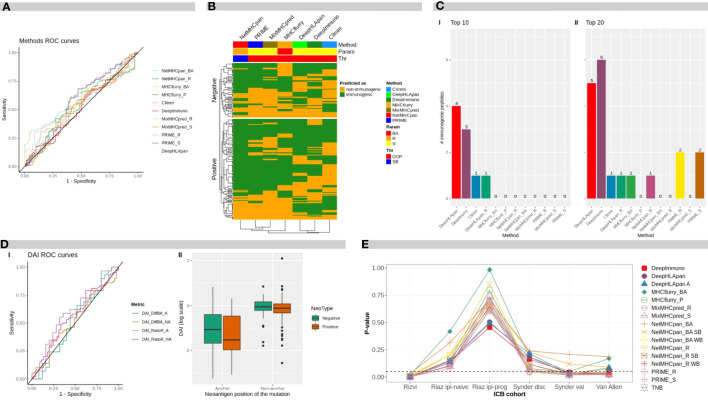
Benchmark results. **(A)** ROC curves for different software programs and direct metrics over mutated peptides. **(B)** Heatmap of methods predicted classification over neoantigens. BA, binding affinity; R, rank; S, score; DOP, distance to the optimal point; SB, strong binder. **(C)** Number of immunogenic peptides between: I top 10-ranked peptides, and II top 20-ranked peptides, according to each method. **(D)** I ROC curves for difference DAI and ratio DAI over anchor and non anchor position mutated peptides. II distribution of ratio DAI in logarithmic scale, for anchor and non-anchor position mutated peptides comparing negative and positive immunogenicity peptides. DAI, Differential Agretopicity Index. **(E)** P-values of association between TNB and clinical response to ICB distribution over ICB cohorts for TNB calculated according to each evaluated method. TNB, Tumor Neoantigen Burden; ICB, Immune Checkpoint Blockade.

**Figure 2A** shows the Receiver Operating Curve (ROC) plots for each evaluated method and metrics where the ROC Areas Under the Curves (AUCs) ranged between 0.52 and 0.60 (by using the ITSNdb), suggesting a difficulty in distinguishing immunogenicity over TSNs known to bind to MHC-I for all methods.

In **Table 1**, the performance metrics reached by all methods over the ITSNdb and over the validation dataset are shown. The latter includes 113 non-immunogenic neopeptides-HLA pairs (with unvalidated MHC-I presentation) and 7 immunogenic, non-SNV derived, neoantigens-HLA pairs with both MHC-I presentation and immunogenicity experimentally validated. This dataset emulates a real scenario of NGS derived candidate neoantigens with known true positive cases.

The high heterogeneity observed on Sensitivity (Se), Specificity (Sp), Positive Predictive Value (PPV) and Negative Predicted Value (NPV) performance metrics for each method over ITSNdb as well as the FPR and FNR on the validation dataset, did not allow a clear identification winner or top performer (**Table S1**). Therefore, in order to identify the top performers (i.e best performer in **Figure 1C**) the following total score was built for each method-metric-threshold predictor combination: the average and standard deviation of the ranks (AR and ARsd respectively) of the DOP and the F1-score (calculated on the ITSNdb) and of the FPR and FNR (calculated over the validation dataset). So, the methods in **Table 1** and **Figure 1D** were ordered according to the lowest AR and lowest ARsd resulting DeepHLApan the top ranked immunogenicity predictor, followed by MHCflurry affinity score. The classification thresholds of these methods were the one that minimize the DOP (see Statistical analysis section). The top performers were both Deep Learning (DL)-based methods and the first was specifically developed to predict immunogenicity.

Although DeepHLApan was not the top performer when evaluated over the ITSNdb, surpassed by the PRIME score (**Table S1**), it shows lesser false positive rates. On the other hand, when ranked by false detection rates on the validation dataset, the top performer was DeepImmuno which had the worst performance with ITSNdb; implying that DeepHLApan achieved a balance between classification and prioritization as suggested by the average rank order.

### 2.4 Almost no overlapping among predicted immunogenic peptides was observed between methods

To evaluate the overlapping between prediction results over the ITSNdb, the top performers between each method were chosen based on their performance ranks. In the heatmap shown in **Figure 2B**, neoantigens (immunogenic and non-immunogenic) are represented on rows and methods in columns. Analyzing column dendrograms, two main groups separate immunogenicity predictors from binding predictors, except PRIME that is grouped with binding predictors showing a very similar behavior to MixMHCpred (PRIME is built on top of it). Besides, it can be observed that the immunogenicity classification overlapping is very poor across methods, suggesting that each method uses a different view of the TSNs immunogenicity. This low overlap reflects the variability between immunogenic peptides and explains the difficulty in selection. Only 7 immunogenic neoantigens were predicted as such by all the methods.

### 2.5 False positive rates over TESLA´s non-immunogenic neoantigens

To further investigate the false positive rate, one of the main challenges towards neoantigen clinical application, 9 and 10 mer, non-immunogenic, MHC-I binder neoantigens were extracted from TESLA database (26). Over this negative dataset, with 297 peptides, the amount of neoantigens predicted as immunogenic by each of the software metrics, were calculated. The best performers were score from MixMHCpred with 30% (90) and rank from NetMHCpan with 31% (92) of FP; on the other hand, the ones who predicted the highest FP rate were DeepHLApan with 100% (297) and DeepImmuno with 79% (234) (all of them by using the DOP classification threshold). Suggesting that variability between databases is huge and performance of each method is dependent on the database used. In particular, in this negative dataset, each of the neopeptide was selected through different prioritization pipelines, so if the software looks for the same features, overlapping predictions would be expected.

### 2.6 Prioritizing candidates TSNs

Another widely applied approach (17, 18) to evaluate different neoantigen prediction software is, instead of using a classification cut-off threshold, to select the top ranked peptides from a list of candidate TSNs, usually designated through a somatic mutation discovery process followed by a mutated peptide-MHC-I presentation prediction. The validation dataset, containing 113 non-immunogenic neopeptides and 7 immunogenic neoantigens was used to feed all methods. Then, candidate TSNs were ranked according to the different method scores. In addition, the originally proposed rank method of DeepHLApan (18), which includes peptides with immunogenicity score ≥0.5 and ordered by means of integrative binding prediction across population alleles, was also evaluated.

**Figure 2C** shows the number of true immunogenic peptides that were included in the top 10 and top 20 ranked TSNs for each method (I and II respectively). It can be seen that the best method in prioritizing the immunogenic peptides was DeepHLApan (Immunogenicity score), followed by DeepImmuno identifying four and three out of seven of the immunogenic peptides in the top 10, respectively. DeepImmuno ranked six immunogenic TSNs followed by DeepHLApan with five in the top 20. These results are in accordance with the FNR in **Table 2** as well as the rank over validation dataset highlighting prioritization performance. It is worth noting that the DeepHLApan prioritizer method proposed by its authors (based on an integrative binding score over population alleles) only prioritized one immunogenic neoantigen between the top 10 and top 20 ranked TSNs, suggesting that its immunogenicity score is better for prioritizing TSNs than their proposed rank method to choose TSN candidates for clinical experimentation. Another remarkable fact is that DeepHLApan, DeepImmuno and CIImm (35) developed for predicting immunogenicity instead of binding strength, were the only methods prioritizing immunogenic peptides in the top 10 of the 120 ranked TSNs of the validation dataset.

**Table 2:** P-values of TMB and TNB association with ICB response through clinical cohorts and methods utilized for TNB calculation.

Cohort	Year	Cancer	N° patients	TMB	TNB	Method
Rizvi (30)	2015	NSCL	34	P = 0.0008	P = 0.0018	netMHC WB_BA_ v3.4
Van Allen (31)	2016	Melanoma	110	P = 0.0076	P = 0.028	netMHCpan WB_BA_ v2.4
Synder Discovery (32)	2014	Melanoma	25	P = 0.01	NS	netMHCpan WB_BA_ v3.4
Synder Validation (32)	2014	Melanoma	39	P = 0.009	P = 0.017	netMHCpan WB_BA_ v3.4
Riaz ipi-naïve (33)	2017	Melanoma	33	P = 0.06	NS	netMHC WB_R_ v4.0
Riaz ipi-prog (33)	2017	Melanoma	35	NS	NS	netMHC WB_R_ v4.0

TMB, Tumor Mutation Burden; TNB, Tumor Neoantigen Burden; NSCL, Non-Small Cell Lung cancer; P, Wilcoxon test P-value; WB_BA_, Weak Binder definition using BA < 500nM; v, version; WB_R_, Weak Binder definition using R < 2%; NS, Non-Significant.

### 2.7 Relative binding affinity is not an immunogenicity predictor

The Differential Agretopicity Index (DAI) or relative binding affinity, is a popular, but controversial, concept within the field, developed as an indicator of immunogenicity over anchor positions (positions that allow peptide-MHC-I binding) mutated peptides, which compares BA predictions for WT and mutated peptides pairs (26, 36, 37).

Here, ROC curves and AUCs were calculated for difference-DAI over NetMHCpan BA predictions and ratio-DAI over NetMHCpan R predictions for two different datasets: one containing all mutated anchor position TSNs from ITSNdb, and the other with non-anchor position peptides; resulting in four ROC curves, as shown in **Figure 2D**, panel I.

The best performance in terms of AUC was for ratio-DAI anchor position (0.59), followed by ratio-DAI non-anchor position (0.56). Consequently, **Figure 2D**, panel II shows the distribution of the ratio-DAI for anchor and non-anchor mutation position, between immunogenic and non-immunogenic TSNs. As expected, the non-anchor position DAI is near 1 (same predicted R for mutated and WT pairs) without differentiating between positive and negative TSNs. However, in contrast to previous reports, no significant difference was found between immunogenic and non-immunogenic TSNs according to DAI values (P=0.25).

### 2.8 TNB calculated over TNS predicted as immunogenic were not superior to TMB as an ICB biomarker

Tumor Neoantigen Burden (TNB) has been suggested as an ICB biomarker as the Tumor Mutation Burden (TMB) (5, 38). Originally, the TNB is derived from the TMB by counting those neoantigens predicted to be bound to the MHC-I molecules by BA predictors. Here, the TNB was calculated by using TSNs predicted as immunogenic by each method, presented in **Table 1**, on each subject from six clinical cohorts from four different studies (30–33). **Table 2** shows the P-value (Wilcoxon test) of the difference between responders and non-responders to ICB for TMB and TNB from the original publication.

TNBs calculated with benchmarked methods were evaluated through their association with clinical response. **Figure 2E** shows the P-values of the TNBs contrasted between the different outcomes of each clinical cohort presented in **Table 2**. It can be seen that the original observed association between TNB and outcome loses significance upon the immunogenicity predictor used; meanwhile TMB, according to **Table 2**, resulted in significant differences in differentiating responder vs non-responders in all cases except for the Riaz ipi-prog cohort. This suggests that, despite immunogenicity predictors diminish the amount of candidate TSNs, which may result in being positive for vaccine development, the derived TNB may result in an inappropriate ICB biomarker, which may be impacted by the fact that most of the predictors resulted in high false discovery rates.

## 3 Discussion

It has been stated that not every MHC-presented peptide is an immunogenic T cell epitope (39) and even WT peptides have also been found bound to an MHC-I complex (40), being binding affinity a necessary but not sufficient condition to exert an immune response. It has also been suggested that the binding strength of immunogenic neoantigens should neither be too strong nor too weak. Moreover, it has been argued that a constant and strong binding may induce T cells exhaustion and peripheral tolerance (41). Another biological process involved is central tolerance during negative selection of self-reactive T cells in thymic development. It may be responsible for the lack of recognition of p-MHC complex by TCR, as a result of the deletion of cross reactive TCRs between WT and mutated peptide; this may occur due to their high sequence similarity, which is what makes neoantigens different from pathogens epitopes (30, 41). These mechanisms support that binding affinity strength seems not to be a decisive factor in cancer context. Thus, in order to evaluate neoantigen immunogenicity predictors, it turns crucial to account for neoantigens with experimental validation of the whole processes (i.e. cell processing, MHC-I binding and TCR recognition). This goes in line with current efforts focused on deciphering what makes a single amino acid change lead to immunogenic neoantigen recognition by TCR.

In this work, to overcome the aforementioned limitation, the Immunogenic Tumor Specific Neoantigen database - ITSNdb was created. A new database with experimental validation of positive cell processing and peptide-MHC-I binding and positive/negative immunogenicity, thereby encompassing both non-immunogenic and immunogenic neoantigens that are known to bind to MHC-I molecules. Consequently, allowing the assessment of immunogenicity prediction. Then a comprehensive evaluation of seven state-of-the-art methods for predicting neoantigen immunogenicity was performed. Three of them, MHCflurry, NetMHCpan and MixMHCpred, were developed to predict the binding affinity between the peptide and MHC-I molecules, which is considered as the first step for the identification of candidate peptides. For the second step, peptide immunogenicity has been assessed based on features such as binding strength, neoantigen relative abundance and sequence similarity to viral epitopes (e.g. foreignness), among others (26, 42). In this scenario, binding affinity predictors were used for both neoantigen definition and immunogenicity classification/prioritization. The other four methods were developed to predict immunogenicity; PRIME and DeepHLApan being specific for neoantigen prediction unlike CIImm and DeepImmuno (developed to predict general immunogenicity). With ITSNdb only positive vs negative immunogenicity could be evaluated in a fairly manner, leaving binding affinity performance out of the scope of the present study.

The evaluation revealed that none of the methods could be considered the best immunogenicity predictor, since they show very low AUCs and loss of Specificity in favor of Sensitivity. Therefore, to sort predictors through fair criteria, the average and standard deviation of the ranks order of DOP and F1-score considering ITSNdb and the ranks order of the FPR and FNR over a validation dataset was used. This ordering criteria resulted in DeepHLApan as the best performer predicting immunogenic neoantigens. Notoriously, the only DL method created specifically to predict neoantigen immunogenicity. PRIME score with DOP threshold, was the best in classifying immunogenic vs non-immunogenic neoantigens (also developed to predict neoantigen immunogenicity), a desirable characteristic for the development of new biomarkers. DeepImmuno was the best over the validation set, predominantly due to its capability in recognizing negative peptides and reducing FPR, an essential requisite for vaccine development. DeepHLApan achieved a balance between those characteristics, and was the best at prioritizing positive peptides between the ranked top 10 predicted TSNs based on its immunogenicity score which resulted superior against their originally proposed prioritization method based on an integrative binding affinity across allele population rank score (18).

Surprisingly, most of the methods, in particular DeepHLApan, provide high FPR when evaluated over TESLA negative examples. This may happen since all the TESLA candidate neoantigens were previously selected as immunogenic by means of in-silico prioritization pipelines but resulting non immunogenic by posterior experimental validation. This may imply that current software methods require well curated neoantigens with proven positive/negative immunogenicity, as proposed with the ITSNdb selection protocol, among other features, to train them.

Immunogenicity predictions were not shared between methods over the ITSNdb, meaning that each method takes into account different peptide characteristics; which could be explained by the prediction purpose (binding vs immunogenicity) and by the way each method codified peptides amino acid sequences to feed the model. A clear example is that DeepImmuno codified the peptide through an AAindex (amino acid index) accounting for physicochemical properties with a principal component analysis encoding strategy, whereas DeepHLApan uses a one-hot encoding strategy to codify the peptide amino acids.

Our findings indicate that relative BA over neoantigens with anchor mutations fails in detecting immunogenicity, in opposition to previous reports that assume as immunogenic those neoantigens whose WT counterpart was predicted as no binder, since no tolerance process would have been carried against it.

The effects of classifying TSNs to be used for TNB calculation and biomarker definition or responders’ vs non-responders’ association was inconsistent and method-dependent. They never outperform the TMB. Thus, TMB is still a superior biomarker than TNB. A more accurate neoantigen prediction would be necessary to overcome TMB in predicting ICB response.

Our results suggest that the neoantigen sequence holds information to define its immunogenic potential based on that DL methods performance is better than random but improvements are still needed. To achieve this, appropriate training datasets are required. Based on the evidence that WT peptides can be presented on the surface (40) and that they may exert a weak, *in-vitro* immunogenic response at high peptide concentration (3) it is suggested that the use of negative examples built by sampling random sequence from human proteome seems an inadequate approach as well as the use of immunogenic peptides from pathogens as positive examples (28) for training neoantigen immunogenicity predictors. ITSNdb pretends to fulfill that requirement, because of two main characteristics: immunogenic neoantigens with experimentally proven processing and presentation as positive dataset, and non-immunogenic neoantigens known to be bound to MHC-I molecule as negative dataset. Present weaknesses are: the current low amount of peptides, expected to increase with the addition of new validated neoantigens in future ITSNdb versions and technical limitations in experimental assays.

It is recommended that DL models developed to predict specifically neoantigen immunogenicity should be further explored jointly with new molecular, patient specific data and objective vaccine responses from clinical trials to enhance their practical application in a clinical environment and the use of the proposed ITSNdb as well as the evaluation algorithm presented here for fair and comparable immunogenicity predictors evaluation.

## 4 Materials and methods

### 4.1 The ITSNdb database

The neoantigens were first collected from PubMedTM using “neoantigen’’ or “neoepitopes” as keywords. The resulting publications were manually curated. Those neoantigens whose inclusion criteria were explicitly described were included in the new database. Next, references from the primary publications (i.e., those that were found using the keywords) were carefully revised. If additional peptides that met the defined criteria were found in these references, the curation process was repeated. The references of the neoantigens from DbPepNeo, CAPD and IEDB were also examined and curated. At the end of this process, neoantigens from 45 scientific publications were included from more than 70 revised manuscripts.

### 4.2 Validation data set for false detection rate evaluation and simulated prioritization scenario

In order to simulate a real scenario of TSN prioritization (i.e. selecting candidate TSN to be included in a vaccine to evaluate their immunogenicity), we merge two TSNs candidates lists. The first one containing 109 neopeptides, 4 with 2 HLA associations, resulting in 113 non-immunogenic neopeptides with experimental negative immunogenic assay of 9-mer length derived from SNVs (3), originally selected by their predicted binding affinity (NetMHCpan version 4.0) and not included in the ITSNdb due to missing binding affinity experimental validation. The second one holds 6 non-SNV derived neoantigens of 9-mer length (2 derived from gene fusion events, 3 originated from intronic retention and 1 derived from 2 nucleotide deletions resulting in a new open reading frame), one of them with 2 HLA-associated restriction alleles resulting in 7 neoantigens with validated MHC-I presentation and immunogenicity (1, 2, 4); leading to a 120 peptides-HLA pairs dataset (available at github/elmerfer/ITSNdb).

### 4.3 TESLA negative dataset

TESLA dataset has a total of 608 neopeptides, all of them were in-silico prioritized by different research teams (26) and chosen for experimental validation. All of them were tested for MHC-I binding and immunogenicity; The reason why it could not be incorporated into ITSNdb is the lack of information about WT sequence or gene of origin into the public database; immunogenic neoantigens were excluded from the analysis because processing and presentation were not validated. In order to be used as a tool for FP evaluation, some filters were applied and only non-immunogenic neoantigens with a sequence length of 9 or 10 amino acids and a measured binding affinity< 500 nM were retained. On the other hand, these non-immunogenic neoantigens were not included in the validation dataset, since it is not possible to reproduce such neopeptides selection in a real clinical setting; consequently TESLA negative dataset was not included in the evaluation algorithm either.

### 4.4 ICB TMB cohorts and TNB evaluation

Four ICB treated cohorts (see **Supplementary Information**) (30–33), with TMB evaluation, were used to calculate TNB according to each evaluated software. These datasets provide the list of candidates TSN predicted to bind to the MHC-I complex (available at github/elmerfer/ITSNdb).

### 4.5 Immunogenicity predictors

To evaluate the performance of current methods and metrics to assess neoantigen immunogenicity, seven state-of-the-art types of software (**Table 3**) were run using the authors’ default preferences over ITSNdb.

**Table 3:** Software description.

Software	Category	Method	Platform	Ref	URL
netMHCpan 4.1	MHC-I Binding	ANN	WEB/C++	Reynisson (13)	https://services.healthtech.dtu.dk/service.php?NetMHCpan-4.1
MHCflurry 2.0	MHC-I Binding	CNN	Python	O´Donnell (14)	https://github.com/openvax/mhcflurry
mixMHCpred 2.1	MHC-I Binding	Mixture model	C++	Gfeller (15)	https://github.com/GfellerLab/MixMHCpred
PRIME 2.0	Immunogenicity	Logistic regression	WEB/C++	Schmidt (16)	http://prime.gfellerlab.org/
DeepImmunno-CNN	Immunogenicity	CNN	WEB/Python	Li (17)	https://deepimmuno.research.cchmc.org/
DeepHLApan 1.1.1	Immunogenicity	RNN	Python	Wu (18)	https://github.com/jiujiezz/deephlapan
Class I Immunogenicity	Immunogenicity	Linear regression	WEB	Calis (35)	http://tools.iedb.org/immunogenicity/

ANN, Artificial Neural Network; CNN, Convolutional Neural Network; RNN, Recurrent Neural Network.

The most recently updated and widely applied methods were chosen for evaluation. They should be publicly available and cover a wide diversity of HLA alleles.

BA prediction software

NetMHCpan 4.1: is an artificial neural network, designed to predict peptide-MHC-I binding. It uses the NNAlign_MA framework to allow for the incorporation of eluted mass spectrometry (MS) data. Thus, it is trained to estimate both the BA and the eluted mass as ranks. For a peptide-HLA restriction pair, it provides the eluted rank (%R) and the BA (nM) to their restricted HLA (13). The authors propose to classify the peptides as either strong (SB), weak (WB) or non-binders, according to predefined score thresholds (by default, SB=%R ≤ 0.5% and WB=0.5%<%R≤ 2%).

MHCflurry 2.0: is a trained convolutional neural network (CNN) that includes affinity measurements and MS datasets. It predicts BA in nM (lower values indicate stronger affinity and the percentile (P) of the affinity prediction (calculated among a large number of random peptides tested on that allele). The authors do not provide predefined thresholds to outline strong binders, but they suggest using the popular threshold of BA<500 nM or P<2% to differentiate binder from non-binder peptides (14).

MixMHCpred 2.1: is a binding motif deconvolution in HLA-I peptidomic model, trained on mass spectrometry elution data. It provides affinity scores (the higher the score, the stronger the peptide binding to the HLA-I) and ranks (the lower the rank, the stronger the peptide binding to the HLA-I) (15).

Immunogenicity prediction software

DeepImmuno-CNN 1.2: A web application implementing a CNN that predicts immunogenicity of MHC–peptide pairs. It uses MHCflurry to predict BA. The model provides a continuous immunogenic score. The greater the peptide scores, the higher the chance for it to be immunogenic. No score threshold is provided to define immunogenicity (17).

T cell class I p-MHC immunogenicity predictor (CIImm): A web application implementing a model to predict the immunogenicity of new peptides-MHCs complexes. It provides an immunogenicity score calculated as a position-dependent weighted sum of the non-anchor amino acids of the neoantigen amino acid sequence. Higher scores mean a higher chance for the peptide to be immunogenic. No threshold is provided to define peptide immunogenicity (35).

PRIME 2.0: web application implementing a class I immunogenicity predictor combining affinity predictions to HLA-I molecules, performed by mixMHCpred, together with TCR recognition propensity, trained as a logistic regression. Non-immunogenic peptides receive a score of 0 (16). A rank is also provided.

DeepHLApan 1.1: recurrent neural network (RNN) that combines a binding model with an immunogenicity model trained to make predictions about neoantigens. Peptides with an immunogenicity score > 0.5 are considered immunogenic. It also postulates a rank between the input peptides according to the probability of activating T-cells (18).

In addition, the Differential Agretopicity Index (DAI), derived from NetMHCpan predicted BA and R metrics (DAI_BA_=WT_BA_-mut_BA_ and DAI_R_=mut_R_/WT_R_) (36, 37), was also evaluated.

### 4.6 Statistical analysis

All the predicted output values from all the considered methods and their derived metrics were evaluated over ITSNdb through ROC curves (pROC R library) (43) and the AUC.

Since the output of every method are continuous values (i.e. scores, binding affinity scores, binding affinity ranks and binding affinity percentiles), a threshold for each of them should be defined in terms of: if the method`s output value for neoantigen “*z*” in the ITSNdb is greater/lesser (depending on the methods) to some threshold value, then the neoantigen will be classified as immunogenic/non-immunogenic. Despite some authors proposed such classification thresholds (for instance SB, WB for NetMHCpan and score > 0.5 for DeepHLApan), we also define the best threshold for all the methods tested on the ITSNdb by chosen the “z-th” output value_z_ for each method that minimizes the Distance to the Optimal Point (DOP) as the “best threshold” for such method, as follows:

For each threshold_i_ (chosen from the minimum output value_z_ to the maximum output value_z_ achieved on the ITSNdb neoantigens for the specific evaluated method) the Se, Sp, PPV and NPV are obtained and the DOP_i_ calculated.

$DOP_{i}=\left(\sqrt{{\left(Se_{i}-1\right)}^{2}+{\left(Sp_{i}-1\right)}^{2}+{\left(PPV_{i}-1\right)}^{2}+{\left(NPV_{i}-1\right)}^{2}}\right)$ then $Best\;threshold=threshold_{i}=\frac{argmin}{i}\left(DOP_{i}\right)$ as suggested in Fernandez et al. (34).

For each method-metric-threshold (author’s defined and by DOP) the Se, Sp, PPV, NPV and F1 score for positive predictions were calculated. False positive rates (FPR) and false negative rates (FNR) were evaluated through the validation dataset. Then, a rank system was implemented, ranking the 16 methods according to ranks of DOP, F1 (ITSNdb), FPR and FNR (validation dataset). In order to sort the method to define the top performers, the average and standard deviation of all ranked performance metrics were calculated (AR and ARsd). The top performers were chosen as the one having the lowest AR and ARsd. In addition, if only ITSNdb is going to be used, the classification rank (CR) can be obtained as AR and ARsd by just using DOP and F1-score ranks and only use FNR and FPR ranks to calculate the False Discovery Rate Rank (FDRR) when using the validation dataset.

### 4.7 Data and code availability

In order to facilitate the use and availability of the ITSNdb and the compared tools we developed user-friendly R library (ITSNdb, which allow the installation and use of NetMHCpan 4.1, mixMHCpred, PRIME and CIImm) and Colab Python notebooks to facilitate the use of MHCflurry, Deepimmune and DeepHLApan. It also contains all datasets used in this study. All of them are free available at https://github.com/elmerfer/ITSNdb.

## Data availability statement

The datasets presented in this study can be found in online repositories. The names of the repository/repositories and accession number(s) can be found below: https://github.com/elmerfer/ITSNdb.

## Author contributions

GN and EF designed the experiments, implemented the R code and wrote the manuscript. EF initiated and supervised the project. VB, JF and PP implemented python code and performed evaluation tests MG supervised experiments, LP supervised code design, GM and HL supervised the experiments and provided critical advice. GN, EF, VB and JF contributed to the discussion and the processing of experimental results. All authors took part in the discussion and editing of the manuscript. All authors contributed to the article and approved the submitted version.

## Supplementary material

Supplementary Table 1 Performance metrics calculated over all predictive methods.
