# Supplementary material for: Unraveling tumor specific neoantigen immunogenicity prediction: a comprehensive analysis
Source: Front Immunol. 2023 Jul 25;14:1094236. doi: 10.3389/fimmu.2023.1094236 (PMC10411733; doi:10.3389/fimmu.2023.1094236)
Supplement: Supplementary file 2 [file DataSheet_1.docx]

Unraveling tumor specific neoantigen immunogenicity prediction: a comprehensive analysis

Supplementary information

**Supplementary Note 1: Software and algorithms application**

Seven predictive software were run over the 199 peptides from ITSNdb, in order to compare software predictions with validated data. Statistical analysis was performed with R language version 4.2.1.

**Friendly optimized software installation and usage**

netMHCpan:

<https://github.com/elmerfer/ITSNdb>

mixMHCpred:

<https://github.com/elmerfer/ITSNdb>

MHCflurry:

<https://colab.research.google.com/github/elmerfer/ITSNdb/blob/main/Colab/MHCFlurry_Colab.ipynb>

CIImm:

<https://github.com/elmerfer/ITSNdb>

DeepImmune:

<https://colab.research.google.com/github/elmerfer/ITSNdb/blob/main/Colab/DeepImmuno_Colab.ipynb>

PRIME:

<https://github.com/elmerfer/ITSNdb>

DeepHLApan:

<https://colab.research.google.com/github/elmerfer/ITSNdb/blob/main/Colab/DeepHLApan_Colab.ipynb>

**Supplementary Note 2: immunotherapy datasets**

Publicly available datasets from three different publications were collected. ICB response over 34 non–small cell lung cancer patients treated with pembrolizumab, an antibody targeting programmed cell death protein-1 (PD-1) was analyzed by [Rizvi *et al*](https://www.ncbi.nlm.nih.gov/pmc/articles/PMC4993154/); a cohort of 110 patients with metastatic melanoma treated with CTLA-4 blockade and their treatment response association, analized by [Van Allen *et al*](https://pubmed.ncbi.nlm.nih.gov/26359337/); another two subject cohorts with metastatic melanoma treated with anti-CTA4 ICB therapy and their response over 25 and 39 patients, evaluated by [Snyder *et al*](https://pubmed.ncbi.nlm.nih.gov/25409260/); and two more patient cohorts with metastatic melanoma treated with nivolumab (anti PD-1), one cohort of 35 patients previously progressed to ipilimumab (anti CTLA-4) and the other, 33 patients ipilimumab naive; from [Riaz *et al*](https://pubmed.ncbi.nlm.nih.gov/29033130/). All datasets contain mutated peptide sequences.

**Supplementary Figure 1**


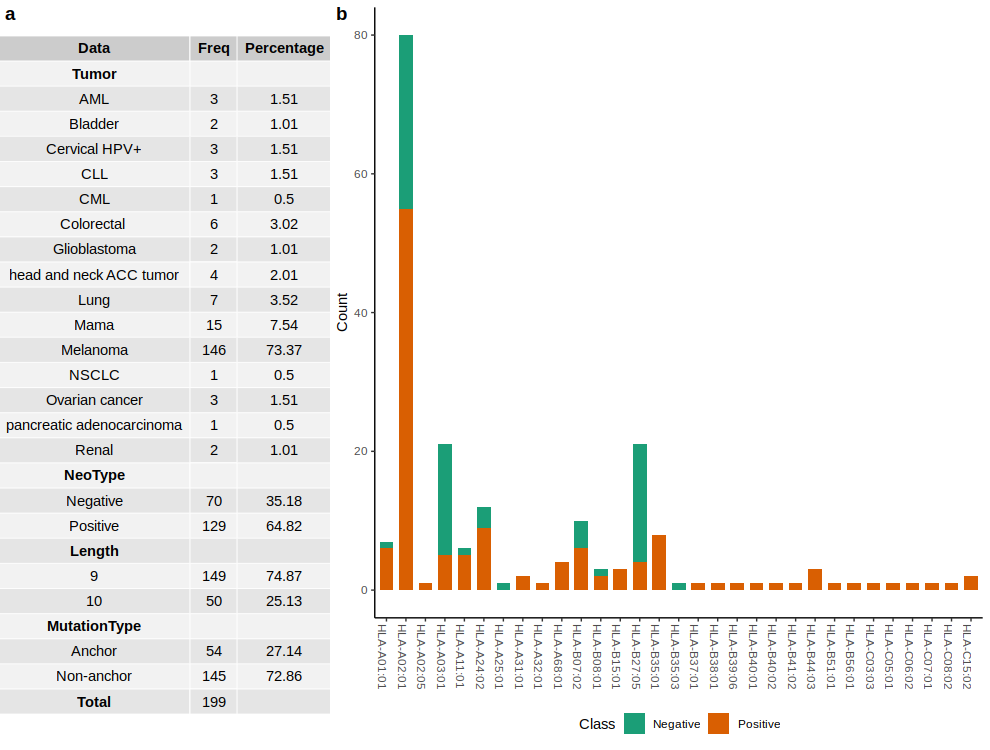


***Sup Fig 1****.* ***Database composition****. (A) Detailed description of the characteristics of neoantigens included in the current database. (B) Distribution of positive (immunogenic) and negative (non-immunogenic) neoantigens by HLA subtype.*
